# Basophil allergen threshold sensitivity, and peanut allergen components in relation to DBPCFC in children with suspected peanut allergy

**DOI:** 10.1186/2045-7022-1-S1-P66

**Published:** 2011-08-12

**Authors:** Caroline Nilsson, Susanne Glaumann, Gunnar Lilja, Magnus Borres, SGO Johansson

**Affiliations:** 1 Karolinska Institutet, Dept. of Clinical Science and Education, Stockholm, Sweden; 2 Sahlgrenska Academy of Gothenburg University, Dept. of Paediatrics, Gothenburg, Sweden; 3 Karolinska Institutet, Dept.of Medicine, Clinical Immunology and Allergy Unit, Stockholm, Sweden

## Background

A few fatal reactions occur every year due to IgE-mediated food allergy among children and teenagers, but there are considerably more near-fatal incidents. To diagnose and give a prognosis as to who will react severely or mildly is difficult for clinicians to assess.

## Aim

To relate the basophile allergen threshold sensitivity (CD-sens) to double blind placebo controlled food (peanut) challenge (DBPCFC) outcome. Further to relate the concentration of IgE antibodies to peanuts and its components to the DBPCFC results.

## Methods

DBPCFC was performed with increasing concentrations of peanut allergen (1 mg to 5 g of peanut) in 42 children with suspected IgE-mediated peanut-allergy. Blood samples were taken for analyses of CD-sens and quantification of IgE-antibodies to peanut and Ara h 1-3, 8-9. Basophils were stimulated in vitro with peanut allergen in descending doses until the threshold sensitivity was reached. CD-sens was defined on the basis of the allergen dose giving 50% of maximal basophil response, measured as expression of CD63. A positive challenge was defined as objective allergic symptoms.

## Results

Among the children, 27 responded with objective allergic symptoms and 15 did not react. The IgE levels to Ara h2 were significantly higher in children reacting to peanut compared to children who did not react at DBPCFC. Negative challenges correlated with high serum levels of IgE-antibodies to Ara h 8. All children with positive challenge, except one (challenge results were difficult to interpret), were positive in CD-sens and among the children non-reacting at challenge, all except one, were negative in CD-sens.

## Conclusions

CD-sens using peanut allergen seems to correlate with the outcome from DBPCFC. High levels of IgE-antibodies to Ara h 2 correlates with clinical reactions to peanut in DBPCFC. Children with IgE to peanut but negative DBPCFC seems to have high levels of Ara h 8 specific IgE-antibodies. CD-sens and component resolved diagnosis may be useful tools in predicting peanut allergy.

